# Anthropization and host habitat influence the abundance of Dermanyssoidea and Trombiculoidea in northwestern Mexico

**DOI:** 10.1007/s10493-025-01005-x

**Published:** 2025-02-12

**Authors:** Angel Herrera-Mares, Oscar Rico-Chávez, Roberto I. Márquez-Hernández, Adriana M. Fernández-González, Andrea Chaves, Carmen Guzmán-Cornejo, Gerardo Suzán

**Affiliations:** 1 Posgrado en Ciencias Biológicas, Ciudad Universitaria, Unidad de Posgrado, Edificio D, 1° Piso, Circuito de Posgrados, Coyoacán, 04510 CDMX México; 2 Departamento de Etología, Fauna Silvestre y Animales de Laboratorio, Facultad de Medicina Veterinaria y Zootecnia, Universidad Nacional Autónoma de México, Coyoacán, CDMX México; 3 Centro Nacional de Innovaciones Biotecnológicas (CENIBiot), CeNAT, CONARE, 1174-1200 San Jose, Costa Rica; 4 Laboratorio de Acarología, Departamento de Biología Comparada, Facultad de Ciencias, UNAM, Coyoacán, CDMX México

**Keywords:** Dominance, Heteromyidae, Index of relative anthropization, Neartic, Prostigmata, Terrestrial rodents

## Abstract

The factors influencing the distribution and abundance of ectoparasites in vertebrates have been thoroughly examined in rodents, particularly concerning ticks and fleas. However, there is a paucity of knowledge regarding mites. The aim of this study was to determine if host or landscape traits are responsible for the abundance of dermanyssoid and trombiculoid mites. Rodent captures were carried out in the northwest region of Mexico between 2018 and 2022 in order to collect mites of the superfamilies Dermanyssoidea and Trombiculoidea. We generated generalized linear models to determine if rodent characteristics or the Index of Relative Anthropization (IRA) contribute to the relative abundance of Dermanyssoidea and Trombiculoidea. The best generalized linear model for Dermanyssoidea suggests that mite abundance is influenced by the IRA and the terrestrial rodent abundance. There is a relationship between the abundance of terrestrial rodents and dermanyssoid mites. Data show that as anthropization increases, Dermanyssoidea abundance decreases. The model shows rodent abundance, and the IRA are key factors in Trombiculoidea abundance. Our findings indicate that trombiculoid mites increase alongside rodents but decline as the IRA increases. This is the first study to use an index to investigate the impact of relative anthropization on Dermanyssoidea and Trombiculoidea in Mexico.

**Supplementary Information:**

The online version contains supplementary material available at 10.1007/s10493-025-01005-x.

## Introduction

Rodents act as the primary reservoirs for a wide range of zoonotic diseases, and they also serve as carriers for a great variety of ectoparasites (Morand et al. 2019). This mammalian taxon harbors a variety of associated mites, including those of the Dermanyssoidea (Parasitiformes) and Trombiculoidea (Acariformes) superfamilies. Dermanyssoidea include families and genera that are primarily facultative nidicolous parasites (Dowling 2006), while Trombiculoidea are protelian parasites that feed on the surface of the host epidermis (Shatrov and Kudryashova 2006). These feeding habits lead them to be considered vectors of some pathogens such as viruses, helminths, protozoa, and bacteria (Herrera-Mares et al. 2022a). The two mite taxa exhibit disparate ecological characteristics, which manifest as distinct infestation patterns. On the one hand, larvae of the Trombiculoidea taxon depend on vegetation that provides moisture to avoid desiccation. This includes, for example, abandoned plantations, tall grasses, and riparian vegetation or vegetation near water bodies (Chen et al. 2022). As a result, their infestation relies on hosts maintaining contact with vegetation. In contrast, Dermanyssoidea depend more on intrinsic host factors, such as taxonomic identity, host community composition, and the landscape configuration in which they exist (Kaminskiené et al. 2020; Krasnov et al. 2020). Nonetheless, as far as we are aware, the impact of host habitat (such as terrestrial, semi-aquatic, arboreal, etc.) on the infestation of this group of mites remains unassessed.

Furthermore, changes in land use impact the dynamics and transmission of rodent-borne diseases (Mendoza et al. 2020; García-Peña et al. 2021). In disturbed sites, the abundance of rodent species that act as reservoirs of zoonotic pathogens increases while the abundance of non-reservoir species decreases (Mendoza et al. 2024). Recently, Gil-Fernández et al. (2024) found no difference in ectoparasite abundance between disturbed and undisturbed sites in the state of Michoacán in southwestern Mexico.

In northern Mexico, studies on mites associated with rodents have been carried out, although all of them have focused only on taxonomy (Herrera-Mares et al. 2022b). The objective of this study was to evaluate whether the abundances of dermanyssoid and trombiculoid mites are being explained by host ecology (i.e., rodent richness, abundance, and habitat) or by landscape characteristics, i.e., the degree of anthropization, measured by an index of relative anthropization. We hypothesize that the abundance of both mite taxa will decline as the index of relative anthropization increases. This decline will be a synergistic effect of host ecology and landscape characteristics. Therefore, we predict that Dermanyssoidea is influenced by fossorial and terrestrial rodents, whereas Trombiculoidea is more closely associated with semiarboreal and terrestrial rodents.

## Materials and methods

### Rodents capture and mite collection

**Fig. 1:**
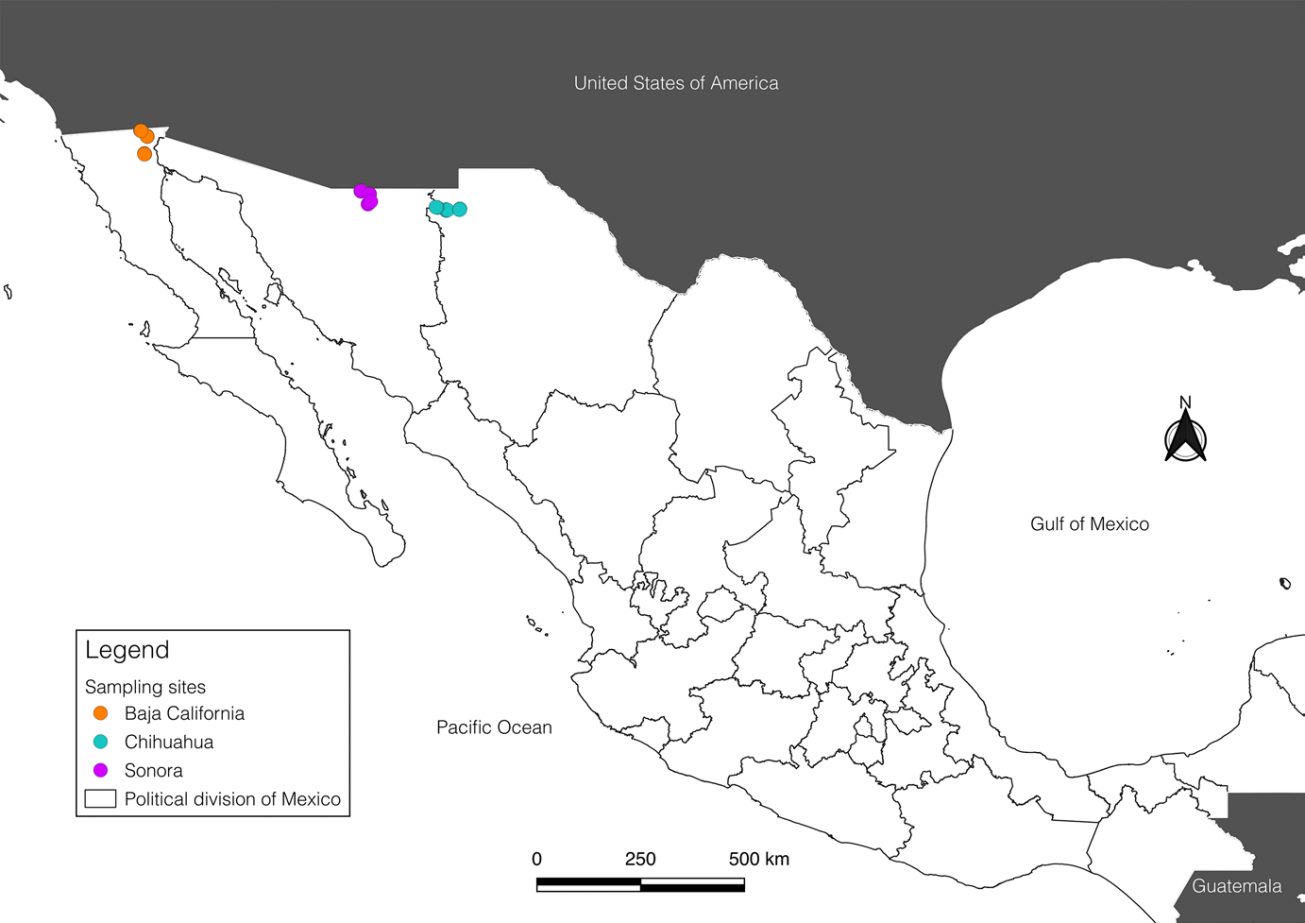
Location of the Mexican states where the study was carried out

Rodents were captured from 2018 to 2022 at sites in three Mexican states adjacent to the northwestern border of the United States: Baja California, Chihuahua, and Sonora (Fig. 1). All individuals were captured using Sherman live traps (7.6 × 8.9 × 22.9 cm; H. B. Sherman Traps, Inc., Tallahassee, Florida, USA), in four 7 × 7 grids placed at 10 m apart, during three consecutive trap nights, with a bait consisting of a mixture of oatmeal, vanilla extract, and peanut butter. For each host were recorded the species, sex, and weight. Rodents were brushed individually for a period of three to five minutes on a white tray containing alcohol and recovering mites using fine-tipped forceps. Rodents were identified using the field guide provided by Reid (2006). Given the environmental conditions, the utilization of anesthetics was not a viable option due to the high evaporation rate. However, the brushing time for each rodent was sufficiently covered. All mites were fixed and preserved in microtubes with 96% ethanol (Guzmán-Cornejo et al. 2020). In the laboratory, they were separated into groups (Dermanyssoidea and Trombiculoidea) and counted under a stereomicroscope (Stemi 508, Zeiss). A subset of specimens was selected for mounting, with the selection criteria based on morphotype. In the case of Dermanyssoidea, an incision was made in the posterior part of the idiosome using an entomological needle. Subsequently, the specimen was placed in a microtube containing a drop of lactophenol, which was then heated with a thermoblock at 70 °C for a period of two hours. In the case of the Trombiculoidea, the same puncture was performed, but the specimens were rendered transparent in a drop of lactophenol in a Petri dish at room temperature overnight. The transparent specimens were then transferred from the lactophenol to a slide, where they were mounted with a drop of Hoyer’s medium (Guzmán-Cornejo et al. 2020). The mite families were identified using taxonomic keys specific to their group, either Dermanyssoidea (Bassols 1975) or Trombiculoidea (Brennan and Goff 1977; Hoffmann 1990). For Dermanyssoidea, we followed the classification of Dowling and OConnor (2010) and Beaulieu et al. (2011), considering Haemogamasidae, Hirstionyssidae, and Laelapidae as separate families; for Trombiculoidea, we followed the work of Nielsen et al. (2021). The mite specimens presented in this study are part of a doctoral project. A separate article will publish the results of the taxonomic identification below the superfamily level, the interactions with rodent hosts, and the search for pathogens. For this particular manuscript, we decided to work at the level where all mites share the same ecological characteristics. Specimens will be deposited at Colección de Ácaros del Laboratorio de Acarología, Facultad de Ciencias, Universidad Nacional Autónoma de México (LAFC-UNAM).

### Land-use characterization

An index of relative anthropization (IRA) was constructed for each site and year of capture to quantify the human influence on the landscape in the immediate surroundings of the site (Martínez-Dueñas 2010; Maya-Badillo et al. 2020). Landscape metrics (i.e., vegetation types, bare surface, water, cropland, construction land, etc.) were calculated within a 100 m radius using the coordinates of the grid midpoint (Morand et al. 2015).

The land use/land cover (LULC) used in this study is from the ESRI Land Cover project (https://livingatlas.arcgis.com/landcover/), which is derived from ESA Sentinel-2 imagery at 10 m resolution. The data provides a map that includes nine surface land use classes, such a vegetation types, bare surface, water, cropland, construction land, etc. (Karra et al. 2021). To facilitate the analysis, we combined and verified the current land use situation and the observations at field and reclassified the data into five classes: water, agricultural land, urban landscape, rangeland, and conservation areas. Values were assigned to each of the classes according to the methodology proposed by Martínez-Dueñas (2010). To consult the value assigned to each of the classes in each of the sampling sites, please refer to Online Resource 1. Each class assigned value multiplies the number of pixels in each buffer to calculate the index. The index ranges from 0 to 1, with 0 representing conserved sites and 1 representing anthropized sites (Martínez-Dueñas 2010; Maya-Badillo et al. 2020). The images were processed with the QGIS software.

### Ecological analyzes

To determine whether rodent traits or the IRA explain the abundance of Dermanyssoidea and Trombiculoidea in rodents, we developed generalized linear models. Rodent host traits included habitat (semiarboreal, terrestrial, and fossorial), species, and the relative abundance of rodents. Host habitat was identified through a synthesis of the research conducted by González-Salazar et al. (2014) and specialized literature on the biology of each species (e.g. Paulson 1988; Mantooth and Best 2005; Ceballos 2014).

In order to ascertain the distribution that best fitted the abundances of Dermanyssoidea and Trombiculoidea, we employed the Akaike information criterion (AIC), as well as the LogLikelihood value. We tested the Poisson and Zero-Inflated Poisson distributions which are commonly used for parasite and mite counts (e.g. McVinish and Lester 2020; Yesilova and Kaki 2016; Márquez et al. 2024). To choose the best model, we relied on the model with the lowest Log-likelihood and degrees of freedom. Ultimately, to validate the selected model, we report the root mean squared error (RMSE) value and obtain the coefficients of the model, along with a graph showing the actual distributions versus the predictions (Bruce and Bruce 2017).

We constructed an Olmstead-Tukey corner test (Sokal and Rohlf 1981) using frequency and rodent host relative abundance to determine dominant, rare, constant, and occasional rodent species. Frequency was defined as the number of IRA values where rodents were captured among the total index values. We performed all analyses using the free software RStudio Version 2024.04.2 + 764 (RStudio Team 2020).

## Results

### Rodents capture and mite collection

**Fig. 2:**
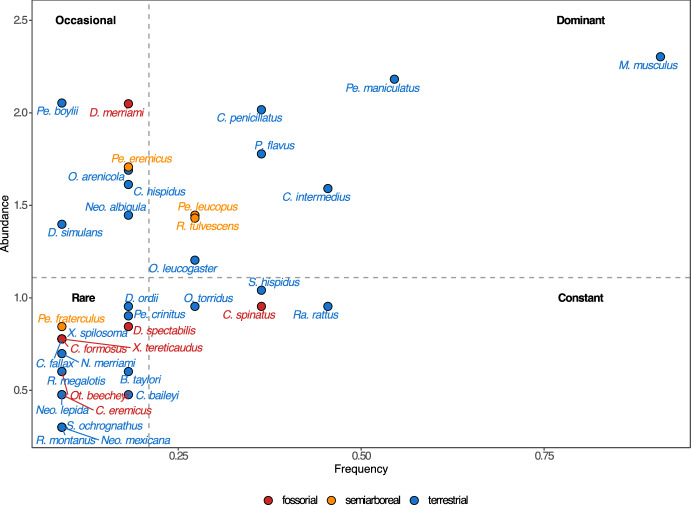
Olmstead-Tukey corner test showing the dominant, occasional, rare, and constant rodent species as well as rodent habitat

We captured 1134 rodents belonging to 37 species in four families: Cricetidae, Heteromyidae, Sciuridae, and Muridae (Online Resource 2). Rodents with the highest numbers of captures were *Mus musculus* Linnaeus, 1758 (Muridae) (n = 201), *Peromyscus maniculatus* (Wagner, 1845) (Cricetidae) (n = 152), *Peromyscus boylii* (Baird, 1855) (Cricetidae) (n = 113), and *Dipodomys merriami* Mearns, 1890 (Heteromyidae) (n = 112). We found a similar rodent composition among the sampled sites (Online Resource 3). The Olmstead-Tukey corner test classified *M. musculus* and *P. maniculatus* as dominant rodent species, and classified *D. merriami* and *P. boylii* as occasional species (Fig. 2).

We recovered 704 mites (596 Dermanyssoidea and 108 Trombiculoidea) from 167 rodents of 22 species, representing a percentage of infestation of 14.7%.

**Fig. 3:**
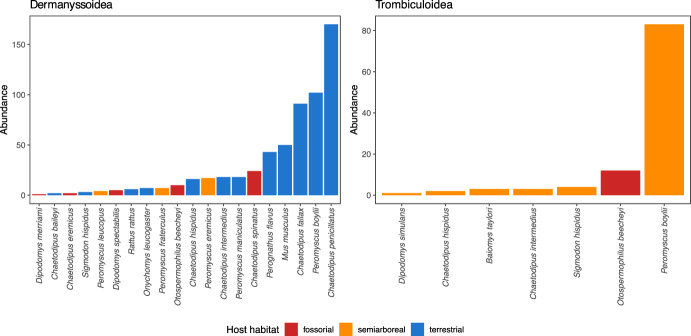
Abundance of Dermanyssoidea and Trombiculoidea collected in this study by rodent species and rodent habitat

We determined 11 taxa belonging to six families: Haemogamasidae, Hirstionyssidae, Laelapidae, and Macronyssidae (Mesostigmata: Dermanyssoidea) and Leeuwenhoekiidae and Trombiculidae (Trombidiformes: Trombiculoidea). Dermanyssoidea were recovered from 20 rodent species, being *Chaetodipus penicillatus* (Woodhouse, 1852), *Chaetodipus fallax* (Merriam, 1889) (Heteromyidae), and *P. boylii,* the rodents with the highest abundance of dermanyssoid mites (Fig. 3). On the other hand, trombiculoid mites were recovered from seven species, being *P. boylii*, *Otospermophilus beecheyi* (Richardson, 1829) (Sciuridae), and *Sigmodon hispidus* Say and Ord, 1825 (Cricetidae), the rodents with the highest abundance (Fig. 3). Both groups of mites coinfested five species: *Chaetodipus hispidus* (Baird, 1858), *Chaetodipus intermedius* (Merriam, 1889), *S*. *hispidus*, *O*. *beecheyi*, and *P. boylii* (Fig. 3).

### Land-use characterization

Our IRA ranges from values of 0 (lightly disturbed sites, characterized by associated vegetation and water bodies) to 1 (completely transformed sites, characterized by built-up areas and crops) (Online Resource 1). We recognized 11 values within this range (Online Resource 3). Baja California had the highest variation in the index with eight values, followed by Sonora with seven and Chihuahua with just three (Online Resource 3). We were able to observe changes in IRA values at some points. For example, in a quadrant in Cananea, Sonora, the IRA went from 0.7 in 2019 to 0.8 in 2022, while in a quadrant in Rancho El Peligro, Baja California, it went from 0.3 in 2020 to 0.5 in 2021 (Online Resource 1). The inability to conduct systematic sampling during the period of social distancing and governmental restrictions imposed by the Mexican government as a consequence of the global pandemic (2019–2021) could plausibly account for these fluctuations in IRA values.

### Ecological analyzes

**Fig. 4:**
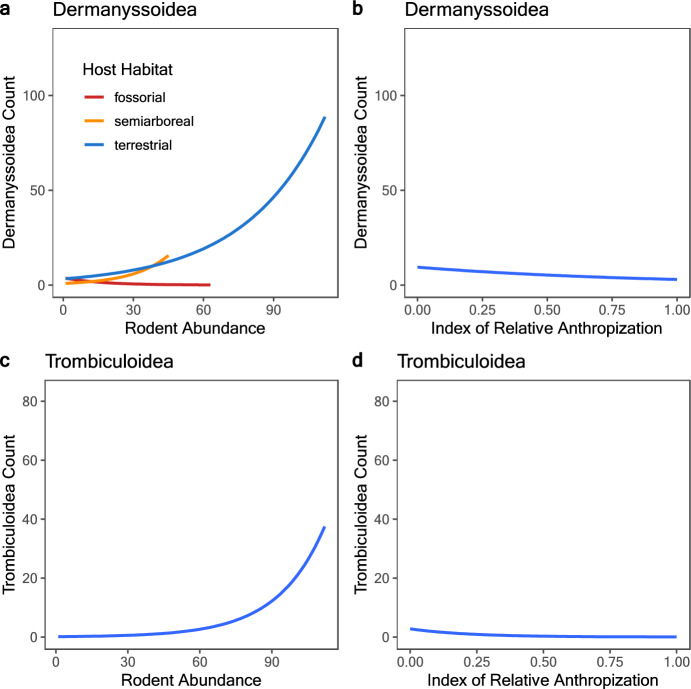
Abundance of Dermanyssoidea showing a positive correlation with terrestrial, semiarboreal and terrestrial rodent abundance (**A**) and negative correlation with the index of relative anthropization (**B**). Abundance of Trombiculoidea showing a positive correlation with rodent abundance (**C**) and negative correlation with the index of relative anthropization (**D**)

The results demonstrated that for both mite taxa, the lower Log-likelihood and AIC values indicated that the abundance data were best represented by a Zero-Inflated Poisson (ZIP) distribution (Table 1). To provide further support for this selection, we also calculated the proportion of zeros. This revealed that 85.71% of Dermanyssoidea abundance values are zeros, while 99.21% of Trombiculoidea abundance values are zeros. Four models were developed that incorporated the IRA along with rodent traits, including habitat (semiarboreal, terrestrial, and fossorial), species, and the relative abundance of rodents for Dermanyssoidea and Trombiculoidea abundances (Table 2). The best generalized linear model for Dermanyssoidea indicates that the abundance of this mite taxon in our system is explained by the IRA, the abundance of rodents and the abundance of terrestrial rodents (Table 3). The abundance of Dermanyssoidea was plotted against the abundance of each rodent habitat (Fig. 4A) and against the IRA (Fig. 4B). The analysis suggested a positive relationship with terrestrial rodents and a negative relationship with the IRA.

**Table 1:** Selection of the distribution model that best fits the abundance data of Dermanyssoidea and Trombiculoidea

Model	Log-likelihood	AIC
ZIP (Dermanyssoidea)*	− 998.1	2000.3
Poisson (Dermanyssoidea)	− 1697.2	3396.4
ZIP (Trombiculoidea)*	− 173.6	351.2
Poisson (Trombiculoidea)	− 643.4	1288.9

*ZIP* zero inflated poisson

*Selected model

**Table 2:** Model selection results for the generalized linear models selected according to the Akaike information Criterion (AIC) and degrees of freedom (df) for the abundance of Dermanyssoidea and Trombiculoidea

Model	Log-likelihood	df
Dermanyssoidea		
IRA + Ab	− 475.9	6
IRA + host species	− 209.3	84
IRA + host habitat	− 537.2	8
IRA + Ab + host habitat*	− 442.8	10
Trombiculoidea		
IRA + Ab*	− 45.11	6
IRA + host species	− 12.23	84
IRA + host habitat	− 87.34	8
IRA + Ab + host habitat	− 34.25	10

*Ab* rodent abundance, *IRA* index of relative antropization

*The model selected

**Table 3:** Estimates of the best generalized linear models for the abundance of Dermanyssoidea and Trombiculoidea in northwestern Mexico

Coefficients	Estimate	Standard error	Z value	p
Dermanyssoidea RMSE: 13.88				
Intercept	1.911	0.159	11.981	< 2e−16***
Ab	0.014	0.001	13.650	< 2e−16 ***
IRA	− 0.780	0.130	− 5.999	1.99e-09 ***
Host habitat – semiarboreal	− 0.198	0.248	− 0.800	0.423
Host habitat – terrestrial	0.922	0.168	5.462	4.71e−08 ***
Trombiculoidea RMSE: 5.67				
Intercept	1.685	0.297	5.667	1.45e−08 ***
Ab	0.024	0.002	8.386	< 2e−16 ***
IRA	− 2.21	0.825	− 2.687	0.00721 **

*IRA* index of relative antropization, *RMSE* root mean squared error

***p < 0.0001, **p = 0.01

**Fig. 5:**
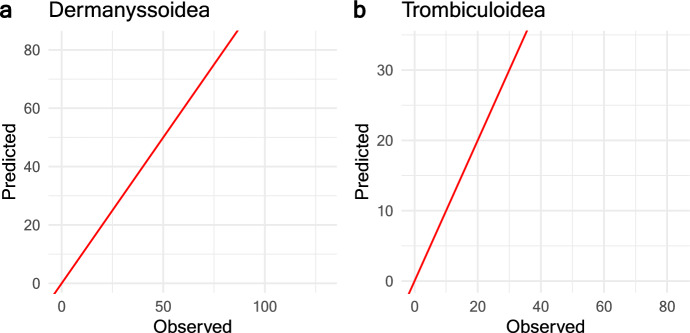
Observed vs. expected values of Dermanyssoidea and Trombiculoidea abundances

The Trombiculoidea model indicated that the primary factors influencing their abundance are the abundance of rodents and the IRA (Table 3) (Fig. 4C and D). The analysis revealed a positive relationship with rodent abundance and a negative relationship with the IRA, emulating the findings observed in Dermanyssoidea. The validation for both selected models is provided by the root-mean-square error (RMSE) values (Table 3), as well as the plot of the observed versus expected values from the abundances for both mite taxa (Fig. 5).

## Discussion

This research was conducted in areas of the country in the northwest where the vegetation is similar, consisting primarily of shrublands and native grasslands with different levels of anthropization degree between sites. Consequently, the rodent species composition is also expected to be similar (Mendoza et al. 2024, Online Resource 3). According to our findings, the most abundant rodent species did not always harbor the most dermanyssoid and trombiculoid mites.

Dermanyssoid mites are nidicolous and it has been hypothesized that the females are responsible for dispersal (Martins-Hatano et al. 2002), leading to reduced host specificity in certain species (Kaminskiené et al. 2023). Nevertheless, a high degree of host specificity has been proposed in other Dermanyssoidea based on a comprehensive examination of the specimens’ morphology, genetics, and ecology (Gettinger et al. 2011). A finer insight into the specificity of the Dermanyssoidea in our system could be obtained through species-level identifications. However, the analyses conducted for this study were carried out at the superfamily level due to the similarity of the ecological characteristics exhibited by these mite species within their superfamilies. This allows for the analysis of the relationship between the total abundance of these mites and the host habitat, as well as the index of relative anthropization.

The rodents with the highest abundance of dermanyssoid mites were *Chaetodipus penicillatus*, *Chaetodipus fallax,* and *Peromyscus boylii* all of which are terrestrial (González-Salazar et al. 2014). The high abundance of dermanyssoid mites in terrestrial rodents suggests that burrows are employed by many sympatric terrestrial rodent species (Lareschi and Galliari 2014; Savchenko et al. 2021). As indicated in the literature, certain rodent species, such as *Chaetodipus baileyi* (Merriam, 1894) and *C*. *penicillatus,* are frequently observed in proximity to one another, given that they inhabit similar microhabitats (Paulson 1988; Mantooth and Best 2005). Our Olmstead-Tukey analysis indicates that *C*. *penicillatus* is a dominant rodent species. Some other species, such as *Mus musculus*, exhibit territoriality and aggressiveness (Crowcroft 1955), which are essential to being dominant in the terrestrial habitat. This dominance may imply that burrows shared by several rodent species are few, making abundance more associated with a particular group of rodents in a particular habitat. The findings corroborate the above, showing that an increase in terrestrial rodent abundance coincides with an increase in Dermanyssoidea mite abundance (Fig. 4A). Conversely, our data indicate that as relative anthropization increases, the abundance of Dermanyssoidea decreases (Fig. 4B). Mendoza et al. (2024) conducted research in the same geographical areas (with the exception of certain localities in Baja California), investigating the response of rodent richness and abundance to a gradient of anthropization. The authors classified the species as zoonotic reservoir and non-reservoir. Their results showed that the abundance of reservoir rodents (such as *M*. *musculus*) increases when anthropization increases, while the abundance of non-reservoir rodents (such as *C*. *penicillatus*) decreases when anthropization increases. It seems that the combined effect of dominance and the terrestrial habitat of rodents is influencing the abundance of dermanyssoid mites in our system.

The larvae of Trombiculoidea, which is the only parasitic stage in their life cycle, inhabit regions characterized by elevated humidity, warmth, and vegetation. This is due to the fact that they depend on these conditions in order to survive (Hoffmann 1990). Furthermore, trombiculoid mites rely on the availability of hosts, a factor that correlates with host abundance. Most of the rodents where we found a higher abundance of trombiculoid mites belong to semiarboreal habitat (Fig. 3), which are also the most likely to have contact with the larvae of these mites. Nevertheless, the model selected did not included the host habitat as an as an explanatory variable for the abundance of the Trombiculoidea. We find that when the abundance of rodents increases, the abundance of trombiculoid mites also increases (Fig. 4C). On the opposite, the abundance of trombiculoid mites decreases as the IRA increases (Fig. 4D). Sites with low anthropization (IRA = 0–0.5) are characterized by the associated vegetation and water bodies (Online Resource 1), which are ideal conditions for finding Trombiculoidea larvae (Chen et al. 2022). It is important to note that the abundance of Trombiculoidea was lower than in other studies conducted in the neotropical zone of Mexico (Guzmán-Cornejo et al. 2020; Barriga-Carbajal et al. 2023; Gil-Fernández et al. 2024). We collected trombiculoid mites by brushing, thereby ensuring standardization in the sampling process. It is plausible that a proportion of other specimens remained attached to specific anatomical locations, such as the axillae. Consequently, the abundance of these mites may be underestimated, and due to the high proportion of zeros, the results should be analyzed with caution.

Our results differ with those of Barriga-Carbajal et al. (2023) and Gil-Fernández et al. (2024) in the Neotropical region of Mexico. These authors reported no differences in the abundance of ectoparasites between disturbed and undisturbed sites (Gil-Fernández et al. 2024), but found that the abundance of rodents and arthropods, including dermanyssoid and trombiculoid mites, increased with the loss of vegetation cover (Barriga-Carbajal et al. 2023). Our findings coincide with Mendoza et al. (2024), who noted that anthropization affected the richness and population of rodents in the dry and semi-arid regions of northwestern Mexico; we observed comparable results regarding the abundance of dermanyssoid and trombiculoid mites. To the best of our knowledge, our study would be the first in Mexico to evaluate the effect of relative anthropization on the abundance of dermanyssoid and trombiculoid mites using an index instead of categorization.

Our initial hypotheses were partially validated for Dermanyssoidea (rodent terrestrial habitat and IRA) and Trombiculoidea (IRA). This work represents one of the few analyses of the ecology of these two large groups of mites, which is not well understood, even for some families such as Haemogamasidae or Hirstionyssidae. A more detailed taxonomic identification would therefore provide additional insights. Nevertheless, this work establishes a foundation for further studies in disease ecology, including pathogen detection and the assessment of additional environmental variables and their relationship with not only the abundance but also the diversity of mites associated with rodents in arid and semi-arid systems, not only in Mexico but throughout the Americas.

## Supplementary Information

Below is the link to the electronic supplementary material.

Supplementary file1 (XLSX 20 KB)

Supplementary file2 (CSV 101 KB)

Supplementary file3 (DOCX 43 KB)

## Data Availability

We declare all data is being provided within this manuscript.
