# Supplementary material for: Anthropization and host habitat influence the abundance of Dermanyssoidea and Trombiculoidea in northwestern Mexico
Source: Exp Appl Acarol. 2025 Feb 12;94(2):37. doi: 10.1007/s10493-025-01005-x (PMC11814010; doi:10.1007/s10493-025-01005-x)
Supplement: Supplementary file 3 — Supplementary file3 (DOCX 43 KB) [file 10493_2025_1005_MOESM3_ESM.docx]

**Figure**: Values of the index of relative anthropization in the three states of northwestern Mexico.

**Figure**: Abundance of rodent species by Mexican State.
